# Identification of neuronatin as a SERCA2b regulin-like protein and assessment of its aggregation propensity via coarse grained simulations

**DOI:** 10.1371/journal.pone.0346335

**Published:** 2026-04-09

**Authors:** Omar Ben Mariem, Lara Coppi, Emma De Fabiani, Ivano Eberini, Maurizio Crestani

**Affiliations:** Dipartimento di Scienze Farmacologiche e Biomolecolari "Rodolfo Paoletti", Università degli Studi di Milano, Milano, Italy; Cinvestav-IPN, MEXICO

## Abstract

Neuronatin (NNAT) is small transmembrane protein involved in a wide range of physiological processes, such as white adipose tissue browning and neuronal plasticity, as well as pathological ones, such as Lafora disease caused by the formation of NNAT aggregates. However, its 3D structure is unknown, and its mechanism of action is still unclear. In this study the two most well-known NNAT isoforms (α and β) were modelled and the interaction with the SERCA2b calcium pump was assessed using computational methods. First, molecular docking identified the same binding region as the one described for phospholamban, a thoroughly described SERCA inhibitor. Then, analyses of the flux of water molecules during molecular dynamics simulations highlighted significant similarities between the behavior of SERCA2b when in complex with phospholamban, and when in complex with either NNAT isoform. These results suggest that NNAT could be considered a “regulin-like” protein. Additional all-atom and coarse-grained simulations of multiple copies of NNAT highlighted a significant aggregation potential of both NNAT isoforms, supporting experimental data.

## Introduction

Neuronatin (NNAT) is a small transmembrane protein encoded in humans by the *NNAT* gene, located on the membrane of the endoplasmic/sarcoplasmic reticulum whose structure is poorly understood. Although initially discovered to be mainly involved in brain development, over the years it has been shown to be involved in a wide range of physiological and pathological processes, e.g., white adipose tissue browning, glucose signalling, neuronal plasticity, and inflammation [1]. The *NNAT* gene is constituted by three exons and two introns, subject to alternative splicing, giving rise to a few isoforms, among which the α and β isoforms, constituted by 81 and 54 residues, respectively, are the most studied. Aberrant NNAT expression is correlated with several diseases, including diabetes, cancer, obesity, neurodegenerative disease, and retina degeneration [2]. Additionally, NNAT aggregates have been found to induce apoptosis, underlying diseases both in neuronal tissue (associated to Lafora disease), and in pancreatic β cells, leading to diabetes mellitus [3,4]. Literature shows that both isoforms α and β can form aggregates in the cytoplasm without difference in propensity. Indeed, this phenomenon appears to be related to the increased expression levels of NNAT and/or proteasome dysfunction in pathophysiological conditions [3,5].

Choi and colleagues [6] recently demonstrated that cold exposure, which physiologically triggers thermogenesis, reduces the expression of NNAT in white adipose tissue (WAT) through ubiquitination and subsequent degradation of the protein. Furthermore, genetic inactivation of the *Nnat* gene in mice promotes browning of subcutaneous WAT stimulating the uncoupling protein 1 (UCP1)-independent thermogenesis.[7,8] This alternative type of thermogenesis is distinguished from the UCP1-dependent thermogenesis because the electron transport chain (ETC) is coupled with oxidative phosphorylation. The ATP produced is then used to fuel a futile cycle of calcium ion (Ca^2+^) transport between the endoplasmic reticulum (ER) and the cytoplasm under the action of the sarco-/endoplasmic reticulum Ca^2+^-ATPase 2b pump (SERCA2b) and the ryanodine receptor 2 (RyR2) [6,9].

SERCA2b is a member of the SERCA pump family, P-type ATPases transporting Ca^2+^ from the cytoplasm to the ER. Three genes, i.e., SERCA1, SERCA2, and SERCA3 are responsible for the production of the 11 isoforms, which share the same function but differ in tissue distribution [10]. They have a fundamental role in regulating Ca^2+^ compartmentalization, necessary for a wide array of physiological processes in all tissues [11–13]. Therefore, the regulation of SERCA activity profoundly impacts cell homeostasis. Phospholamban (PLB) and sarcolipin are two of the key regulators of the activity of SERCA pumps, called “regulins” [14]. These small proteins are present in skeletal muscle fibres and cardiomyocytes, where they inhibit the activity of the various SERCA isoforms, thereby increasing the cytosolic concentration of Ca^2+^ ions and, among other effects, promoting contractions of these fibres [15–27].

Like all pumps, SERCA works by undergoing a series of consecutive conformational changes that results in the ATP hydrolysis and Ca^2+^ transport. It has been proposed that PLB and sarcolipin stabilize one of such conformations, thereby preventing the correct pump function [2,6,28]. It has been recently shown that PLB undergoes phosphorylation at serine residues that negatively affects its inhibitory activity on SERCA2A [23].

NNAT is thought to have a role comparable to the ER-membrane protein PLB, decreasing the transport rate of Ca^2+^ from the cytoplasm to the ER. Interestingly, a recent phosphoproteomic study identified a phosphorylation site on Ser56 of the α isoform, corresponding to Ser29 of the β isoform, which might also suggest a regulatory mechanism similar to that of PLB, by which phosphorylation induces a decrease or complete ablation of the inhibitory effect [29].

In this context, this study aims at proposing a potential interaction mechanism between NNAT and SERCA2b, to characterize the inhibition of the Ca^2+^ pump, which could become a promising potential target for drug discovery. Because of the lack of experimentally solved structures of either NNAT α or β, the first step of the study was the generation of a 3D model for both isoforms. After obtaining a reliable conformation of these structures, it was possible to assess the potential SERCA2b inhibition mechanism, comparing it to the one described for PLB. Additionally, these models were used to study the formation of protein aggregates, involved in neurological and metabolic diseases.

## Materials and methods

### Modeling of human NNAT, SERCA2b, and PLB

Because no experimental structures for human NNAT or SERCA2b were available, modeling procedures were used to obtain reliable 3D structures through the following steps. The sequences of the two human NNAT isoforms (UniProt ID: Q16517) were retrieved from the Uniprot knowledgebase database [30]. A protein BLAST [31] search in the Protein Data Bank (PDB) [32] for a NNAT homolog to be used as a template for homology modeling returned no results for either isoform.

Preliminary secondary structure predictions were run using TMHMM [33] and PsiPred [34]. AI-based *ab initio* strategies were subsequently employed to obtain a reliable model, specifically, RosettaFold [35] and AlphaFold [36].

The BLAST search performed using the sequence of human SERCA2b (UniProt ID: P16615) returned numerous results. Among the available structures that could be used as templates, the *O. cuniculus* SERCA1a structure was selected (PDB code: 4KYT [22]), with an identity percentage of approx. 80% and similarity of approx. 89%, making homology modelling the most appropriate modeling procedure. This specific structure was chosen because it is already in a complex with PLB, making the following comparative steps more reliable. However, the 4KYT structure was lacking the transmembrane helix 11 (TM11), key in the correct functioning of the pump. Therefore, a chimeric approach was employed, modeling SERCA2b on 4KYT for most of the protein, and using 6LLE for the modelling of TM11, obtaining a complete model in the desired conformation. The human PLB model (UniProt ID: P26678) was also built via homology modelling by first superposing the transmembrane helix of the full human PLB monomer (1ZLL [37]) on the PLB structure available in the 4KYT complex, then using that full structure as part of the complex.

### Equilibration and cluster extrapolation

Molecular dynamics (MD) simulations and frame clustering procedures were carried out with Desmond and the Schrödinger Small-Molecule Drug Discovery Suite 2022−3 (D. E. Shaw Research, New York, NY; Schrödinger, New York, NY) [38].

The Desmond System Builder tool was used to place the two obtained NNAT models into a 1-palmitoyl-2-oleoyl-sn-glycero-3-phosphocholine (POPC) membrane bilayer. Protein orientation was set up according to the OPM server, which provides spatial arrangements of membrane proteins with respect to the hydrocarbon core of the lipid bilayer. The system was solvated with SPC water molecules in an appropriately sized orthogonal box (a buffer of 15Å in the x, y, and z dimensions). Sodium and chloride ions were added to reach a 0.15 M concentration and neutralize the system. The system was energy-minimized to relax the assembly and remove clashes between protein, membrane, and solvent in the new setup.

One 1000 ns MD simulation was run for each isoform to obtain equilibrated and more realistic structures. Periodic boundary conditions (PBCs) and the following parameters were set: 300 K and Nose-Hoover thermostat for temperature coupling, 1 bar and Martyna-Tobias-Klein piston for pressure coupling, and 2 fs as the integration time step. Coordinates and velocities of each atom were saved every 0.5 ps. The widely used OPLS4 force field was used to parametrize the atoms for the MD simulations [39].

One additional MD simulation for each isoform was run keeping the protein in solution, to obtain soluble conformations that could be used for the following MD simulations to assess their aggregation potential.

Root mean square deviation (RMSD) and solvent accessible surface area (SASA) were calculated using the Schrödinger Python API. RMSD distances between MD frames were used to perform clustering and obtain a representative structure for both the isoforms.

### Aggregability assessment with all-atom and coarse-grained MD simulations

The most representative medoid of the most populated cluster obtained from the MD simulations run in solution was obtained for each isoform. Two systems were built inserting seven copies of each one in a solvated box in order to obtain a relatively high concentration in a small enough box to observe aggregation in acceptable simulation times. For all-atom MD simulations, 400 ns were run using the previously described parameters.

To obtain the coarse-grained systems, the SIRAH conversion tool available with the AmberTools22 suite was used to convert the groups of atoms in the structures to coarse-grained beads. The SIRAH force field was used to parametrize the systems. MD simulations were run for 1000 ns using the pmemd engine available in the Amber22 suite using Langevin dynamics [40–42]. The aggregation propensity was measured by calculating the number of separate “entities” during the simulation, defined as “a single copy or group of copies not interacting with others”.

### Complexes generation and analysis

The medoid of the most representative cluster for each isoform was extracted from the in-membrane MD simulations and used as starting point for protein∷protein docking calculations using Piper [43]. The procedure was performed using the default Piper parameters, obtaining 10 final poses. No restraints to narrow down the possible interaction interfaces were defined. Because of the high flexibility of the C-terminal portion of both isoforms, only the identical transmembrane helix was considered.

Complexes were also generated using RosettaMPDOCK and the AlphaFold Multimer software, which allows to obtain a further, unbiased confirmation regarding the putative SERCA2b::NNAT binding mode [44,45].

The proteins were then inserted in a POPC bilayer and three replicas of 100 ns MD simulations were run with the same parameters previously described.

The systems with the phosphorylated serine were built by manually adding a phosphate group to the amino acid side chain of the previously described complexes and minimizing the nearby residues before running 3 replicas of 100 ns MD simulations.

The analysis of the interactions between the two proteins and the calculations of the fluxes of water molecules were performed using the Schrödinger Python API and purposefully built python scripts using MDAnalysis [46–48].

## Results and discussion

### Model generation and equilibration

**Fig 1:**
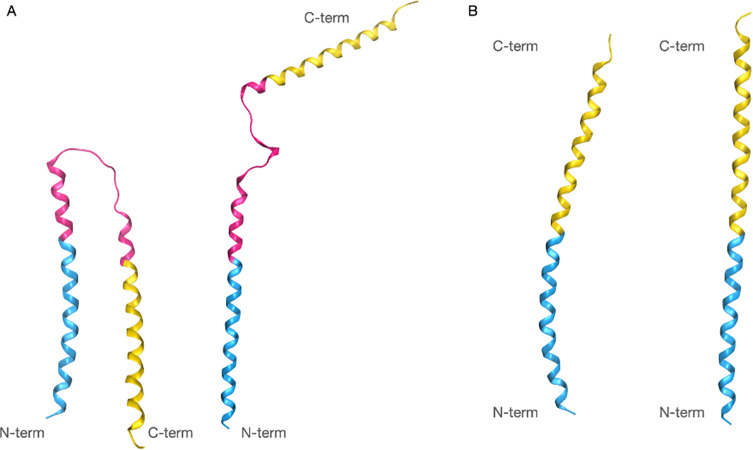
Structure predictions of NNAT α (A) and NNAT β (B) with AlphaFold on the left and RoseTTAFold on the right of each panel. While for the β isoform the two software demonstrate a very similar prediction, the models of the α isoform present very different conformations. However, notably, the two algorithms provide similar predictions of both the helical and unfolded portions. All structures are coloured to reflect the exons in the NNAT DNA (cyan for exon 1, magenta for exon 2, absent in the β isoform, and yellow for exon 3).

As previously mentioned, there seems to be consensus regarding the overall architecture of the two NNAT isoforms [3]. Specifically, both are characterized by a transmembrane helix, an unstructured portion of different length in the two isoforms, and a terminal cytoplasmic helix. Preliminary predictions using TMHMM and PsiPred support these hypotheses (Fig. S1). Interestingly, TMHMM predicts a different orientation of the two isoforms. However, this should be considered as a minor piece of evidence, since the algorithm is mainly built for predicting the orientation of cellular membrane transmembrane proteins, while NNAT can be found in the membrane of the endoplasmic/sarcoplasmic reticulum. The main takeaway from these software should be the secondary structure architecture. The used orientation was determined by the overwhelming agreement of all methods discussed later in this section. Since homology modeling was not possible due to the lack of appropriate templates, *ab initio* methods were applied using AI-based software, specifically RosettaFold and AlphaFold (Fig 1). While displaying significant similarities, AlphaFold predicted an unrealistic conformation for the α isoform (panel A, left side), with the hypothesized cytoplasmic α-helix expressed by the third exon parallel to the N-terminal, which would therefore also be embedded in the membrane. The plDDT scores and PAE maps are reported in SFig.2–3: the highest plDDT values correspond to the predicted transmembrane helix in all models. Additionally, the α isoform model obtained by AlphaFold was the only one presenting outlier backbone dihedral angles values in the Ramachandran plot (Fig. S4). Because of this, to avoid introducing unnecessary bias in the following steps, the models obtained by RosettaFold (with a confidence score of 0.88 and 0.92, respectively) were selected. Notably, the overall predicted architecture strongly resembles the one of PLB and sarcolipin, although in those proteins the transmembrane helix is at the C-terminal.

**Fig 2:**
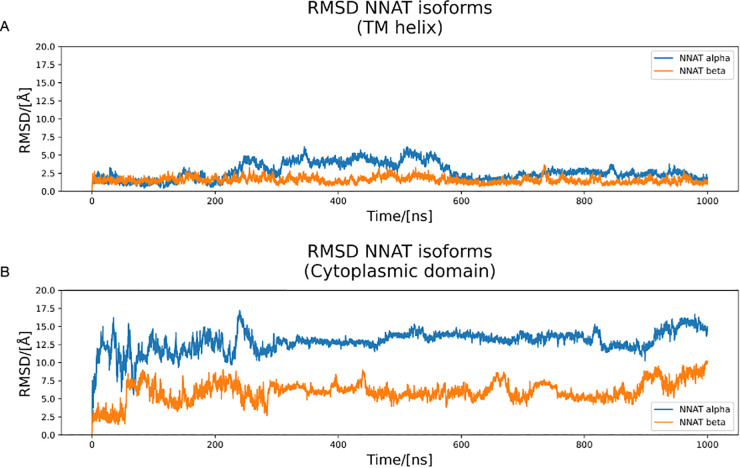
RMSD profiles of the two NNAT isoforms during the MD simulations in the membrane. **A)** RMSD of the transmembrane (TM) α-helices. The profile appears flat with relatively low values, confirming stability of the models in that portion. **B)** RMSD of the cytoplasmic domain after alignment with the transmembrane α-helices. The values of RMSD are high because of the significant relative mobility between the cytoplasmic and transmembrane domain, but the profiles appear flat, demonstrating good overall stability.

As the models could not be considered reliable without further investigations, MD simulations were run to better reflect the physiological conditions and allow the model to relax and find a more stable conformation. Fig 2 reports the RMSD profiles of the proteins during the simulations. It is possible to observe how both isoforms, after an initial significant structural rearrangement (approx. 150 ns), which was expected due to the highly mobile structure, reached a plateau which was maintained throughout the simulations. This is, expectedly, more evident in the transmembrane region, as both isoforms present an α helix.

**Fig 3:**
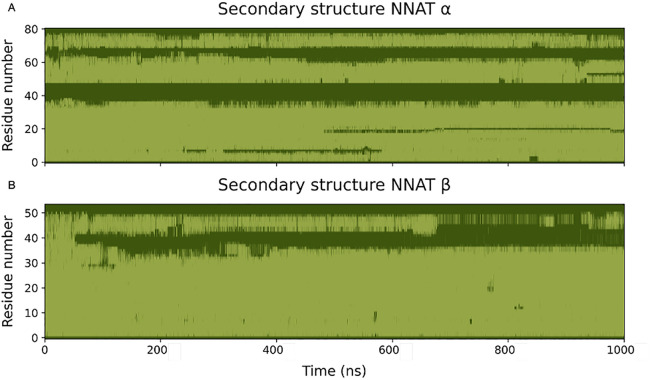
Secondary structure of the two NNAT isoforms. Light green indicates α-helical secondary structures, while dark green indicates an unfolded portion of the protein. **A)** Secondary structure of NNAT α. The model retained a stable transmembrane α helix for the whole simulation up to residue 35. The cytoplasmic portion alternates unfolded portions to α-helical ones. **B)** Secondary structure of NNAT β. The model retained a stable α helix for most of the simulation up to residue 35. Despite the starting conformation as a fully helical structure, most of the cytoplasmic portion loses its secondary structure during the simulation.

The secondary structure analysis confirmed that the largest portion of both isoforms retains the hypothesised α-helical structure, although the large loop in the α isoform, as well as the first 10 residues in the β isoforms are indeed in a random coil conformation (Fig 3). Overall, these simulations indicated that the two isoforms, after an initial structural rearrangement, were stable and maintained a high α helical secondary structure propensity.

**Fig 4:**
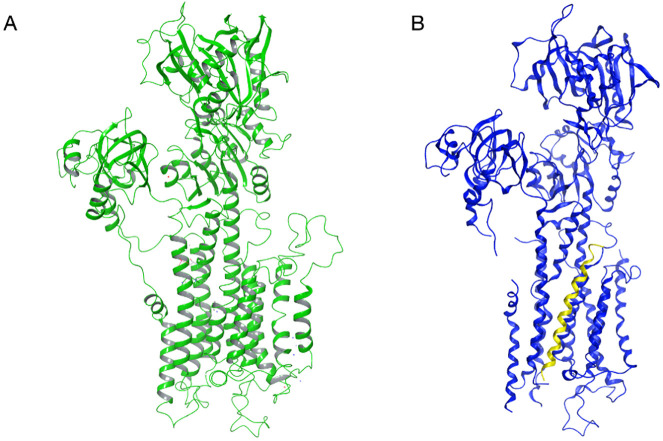
(A) Chimeric 3D model of human SERCA2b and (B) crystal structure (PDB code 4KYT) of the *O. cuniculus* SERCA1b (blue) co-crystalized with its inhibitor PLB (yellow).

To evaluate the mechanism of the inhibition of SERCA2b by NNAT, the calcium pump model was also generated. In this case, a homology modelling procedure could be applied, using a template with an extremely similar sequence from *O. cuniculus* (4KYT, see Fig 4 and alignment in Fig. S5) and an available full human SERCA pump structure (6LLE), obtaining a complete chimeric model. Because the 4KYT template is already in a holo-conformation with PLB, the obtained model was simply energy minimized and no further MD simulations were run on the model to be used for the complex generation step, to maintain the most suitable conformation for protein binding and to avoid bias introduction. A MD simulation of the complex was instead run to assess the water molecule fluxes in the pump, which will be discussed in a following section.

The same structure was used to build a complete human PLB model. The available human PLB experimental structure (1ZLL) was superposed to the truncated structure present in the 4KYT crystal and then merged to the complete chimeric SERCA2b model. The clashing residues were minimized to avoid atom superpositions and prepare the complex for MD simulations to assess the flux of water molecules in SERCA2b, which will be discussed in a later section [3].

### Identification of the SERCA2b::NNAT binding site

**Fig 5:**
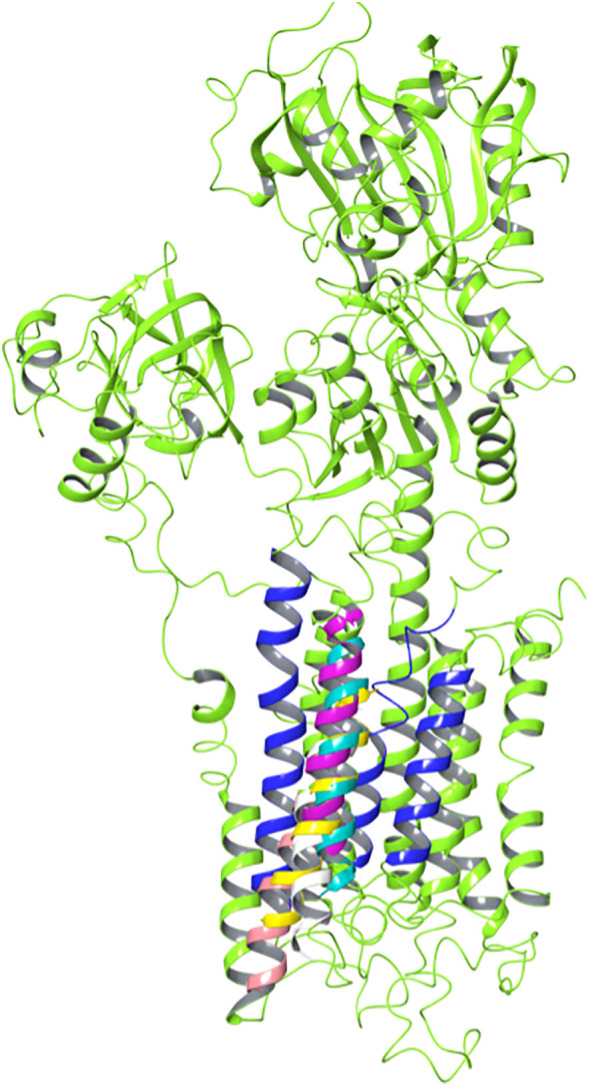
(A) Top ranking protein::protein (PIPER) docking poses. All the represented poses are found in the well characterized M2-M6-M9 groove (colored in the darker blue hue).

After extracting structures of both isoforms from membrane MD simulations, initial protein::protein docking calculations to obtain SERCA2b∷NNAT complexes were carried out. Expectedly, because of the bulky cytoplasmic terminal of both isoforms, and the overall static nature of this procedure, no poses were found. Therefore, to avoid biases caused by the specific frame chosen to extract the structure, only the transmembrane helix (expressed by the first exon, identical in both NNAT isoforms) was used for the calculations. By doing this, several extremely similar poses were obtained, each representing a differently sized cluster of poses (Supplementary Table 1). Considering the 8 most populated clusters (>95% of the total poses), all are located in the groove between helices M2, M6, and M9 of SERCA2b, extensively characterized as the PLB binding site (Fig 5) [15,22,23,49].

The highest‑scoring poses were characterized by the NNAT helix adopting an orientation opposite to that of PLB, with its N‑terminus facing the lumen and its C‑terminus directed toward the cytoplasm. This observation aligns with long‑standing hypotheses suggesting that NNAT C‑terminal tail resides in the cytoplasm, where it could interact with the ATPase and translocation domains of SERCA. Such an arrangement would position NNAT to modulate SERCA activity in similar way to the more extensively studied regulins [50].

Alphafold Multimer was also used to generate models of the complexes in an unbiased way, in order to confirm the obtained results or observe alternative binding modes. In this case, exploiting the *de novo* nature of the modeling procedure, the whole sequences of both proteins were used, as the software will avoid any steric clashes. The results of this modeling procedure for both isoforms can be seen in Fig. S6. The transmembrane helix of both isoforms was predicted to be in the same groove as PLB and as the PIPER docking poses, further reinforcing this site as a very promising putative interaction site.

The structure of the β isoform is coherent with the ones that were generated using both RoseTTAFold and Alphafold, with a fully helical structure, so it was considered a good starting point for MD simulations. On the other hand, the model of the α isoform showed a big portion of the protein embedded in the membrane, which is similar to the one observed in the standalone Alphafold model. Given the much larger cytoplasmic portion of the α isoform, this is not necessarily surprising, as Alphafold lacks understanding of the surrounding environment and is not able to recognize the presence of a membrane around that portion of the model. Therefore, this complex was not used as a reliable starting structure, but the medoid of the previously discussed simulations was used.

To further assess these results, RosettaMPDock was used. This software specializes in membrane protein::protein complexes, so it is able to overcome some of the functional limitations of other programs, such as Alphafold. The best 50 poses can be seen in panel A of Fig S7 and are all extremely similar both structurally and energetically, both among the obtained poses and to the previously obtained results reported in Fig 5. In panel B the cluster analysis of all 1000 poses is reported, which highlights how the majority of them is extremely similar, showcasing two very significant large clusters, and several smaller ones.

The coherent results obtained by this unbiased array of varied software gives a strong and compelling evidence of the putative interaction site between SERCA2b and NNAT being the same as the one with PLB, increasing the credibility of the hypothesis of their similar mechanism of action.

A SERCA2b::PLB complex was also built to act as a control comparison with the NNAT complexes by using the position of the PLB chain found in the 4KYT experimental structure and superposing the complete chain of 1ZLL. MD simulations of all of these complexes, as well as the *apo* SERCA2b model, were performed and further analysed in the following section.

### SERCA2b water fluxes regulation

**Fig 6:**
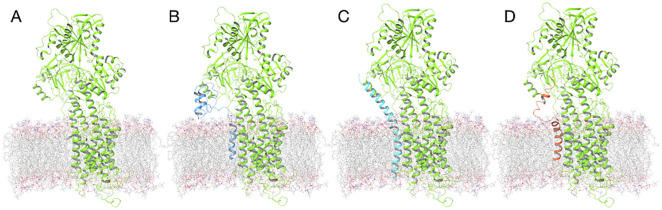
Starting coordinates of the systems prepared for MD simulations. (A) apo-SERCA2b, **(B)** SERCA2b:: αNNAT, **(C)** SERCA2b:: βNNAT, **(D)** SERCA2b::PLB.

Three replicas of MD simulations for each system were performed after inserting the proteins in a POPC bilayer (Fig 6). The data obtained by the apo-SERCA2b system could be considered as a “negative control”, since it simulates how the protein would behave in the absence of its physiological regulators. On the other hand, the SERCA2b∷PLB complex could be considered as the “positive control”, since there is wide experimental evidence regarding its effect on the Ca^2+^ pump.

A quantitative analysis of the water molecules entering and crossing the pump was performed, to assess if and how the interaction with accessory proteins might affect the Ca^2+^ ions pathways across the membrane [51]. While not a perfect correspondence to the movement of Ca^2+^ ions, water molecules can be a useful proxy to sample the pump openings.

**Fig 7:**
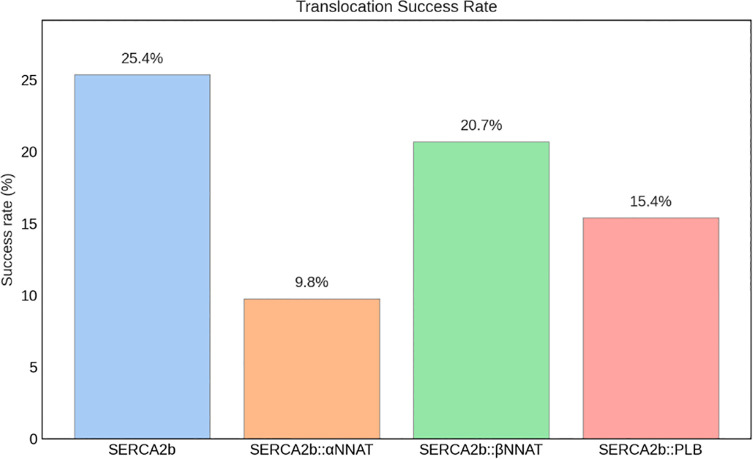
Translocation success rate of water molecules across the membrane in each of the investigated systems during MD simulations.

In Fig 7 the percentage of water molecules able to fully cross from one side to the other of the lipid bilayer over the total number of water molecules entering the volume of the protein are reported. It is possible to see how 25% of molecules in the *apo* SERCA2b system can cross the pump, while this percentage decreases down to 15% in the SERCA2b::PLB system. When interacting with βNNAT the percentage is 21%, demonstrating an intermediate behavior, while it decreases to 10% in αNNAT, apparently causing such a change in the SERCA2b structure that leads to an even greater hindrance to water molecules passage.

**Fig 8:**
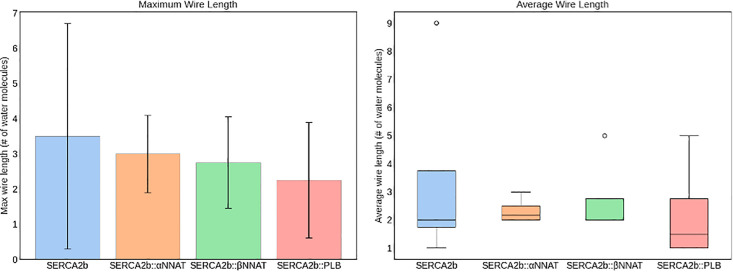
Maximum water wires length (left) and average wire length (right) in SERCA2b during MD simulations.

The formation of water wires was also investigated, as previously done by Espinoza-Fonseca [48]. In particular, we investigated the lengths of water wires as defined by water molecules inside of the protein volume forming a chain of hydrogen bonds. Fig 8 reports the maximum water wire length distribution, on the left, and the average wire length distribution on the right. From the first graph, it is possible to observe that in the *apo* system the longest water wires in each frame are usually longer than when paired with other proteins, with PLB causing the biggest decrease of this measurement. Additionally, by simply looking at the average water wire lengths, the *apo* system demonstrates more often longer wires, with an outlier even reaching 9 molecules. The other systems, especially the ones with NNAT, have a slightly higher median, mostly due to superficial short water wires increasing this value.

A salt bridge was formed for most of the MD simulation between Glu28 of αNNAT and the residue Arg324 SERCA2b known to be important for conformational rearrangements of the M4S4 domain (the salt bridge is present for 74% of the simulation time). On the other hand, the only relevant interactions formed between SERCA2b and βNNAT were hydrogen bonds formed by Trp16 and Tyr17 of NNAT with Ser941 and Asn101 of SERCA2b respectively, which are found in the transmembrane region, rather than the cytoplasmic/endoplasmic regions.

Overall, these results support the hypothesis that the two NNAT isoforms behave similarly to PLB, leading to a decrease in the rate of Ca^2+^ transport across the membrane by causing a specific conformational rearrangement affecting the entry pathways, causing a significant change of the movement of water molecules and their interaction patterns.

### NNAT phosphorylation impacts SERCA2b binding

As mentioned in the Introduction, the structural and functional similarities with PLB may extend to their regulation by the same post-translational mechanism: phosphorylation of a key serine residue, which is known to reduce or abolish PLB-mediated inhibition. To assess whether a similar effect occurs in NNAT, the corresponding serine in both isoforms was phosphorylated, and additional MD simulations were performed.

Analysis of the interactions showed that βNNAT lost both hydrogen bonds upon phosphorylation, whereas αNNAT retained the salt bridge, although its occupancy decreased to 56%.

**Fig 9:**
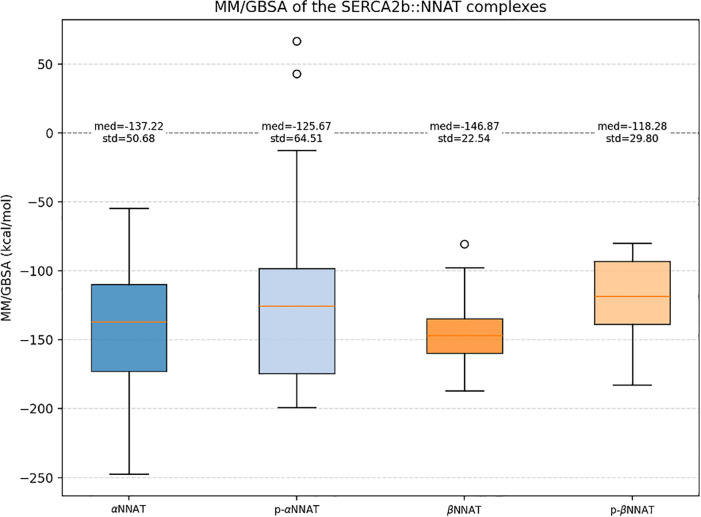
Distribution of the MM/GBSA energy between SERCA2b and the NNAT isoforms. It is possible to see an increase of MM/GBSA values in the systems where NNAT is phosphorylated.

To complement these observations, an energetic evaluation was carried out using MM/GBSA calculations. As shown in Fig 9, phosphorylation led to an overall increase in interaction energy for both isoforms, a trend particularly pronounced in the phosphorylated βNNAT. The complex with phosphorylated αNNAT also displayed two notably high energy values but were much lower in the unphosphorylated NNAT simulations. Lower values correlate with a stronger interaction, therefore an average increase in these values is evidence of complex instability. These results suggest that phosphorylation may function as a similar regulatory process on NNAT as on PLB, although longer MD trajectories and more refined initial conformations may further accentuate the observed differences. Nevertheless, experimental validation remains essential to confirm these interesting, although preliminary, computational findings.

### NNAT isoforms aggregation

Aside from its numerous physiological roles, NNAT has also been found to cause significant cell apoptosis and tissue degeneration in both brain and pancreas due to the formation of toxic aggregates. To assess the aggregation potential of the two NNAT isoforms, MD simulations were run. Initially, a 1 μs long MD simulation of the two models was run in solution, to obtain conformations resembling those adopted by the proteins in the cytosolic compartment, for example, due to overexpression and/or impairment in their degradation pathway. Expectedly, as they were not in their physiological environment, across the membrane, both NNAT isoforms lost some of their secondary structure features and reached a more compact conformation, as seen from the SASA and the radius of gyration (SFig. 8). This is especially evident for the α isoform, while the β NNAT seems to keep a fairly stable level of compactness with small fluctuations over the whole simulation. These significant rearrangements can be seen especially in the first 10 residues of the N-terminal portion for both isoforms, where it is possible to observe a significant loss of secondary structure, as the hydrophobic domain, physiologically inserted in the phospholipid bilayer, unfolded. The conformations of the final frames were used to build systems containing 7 copies of each NNAT isoform, randomly placed in a fully solvated box.

**Fig 10:**
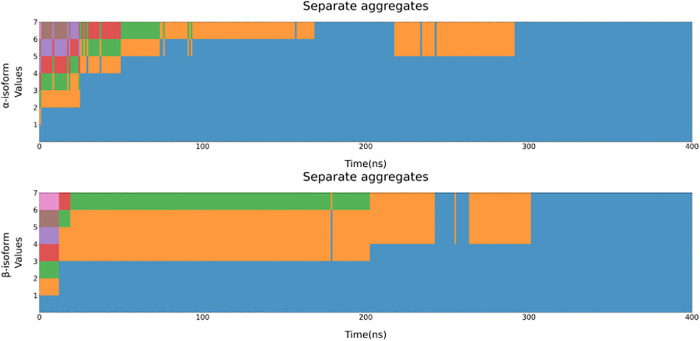
Separate entities observed for the all-atom simulations. Each colour represents a unique “entity”. It is possible to see that, for both the isoforms, copies begin to aggregate rather quickly and after 300 ns all copies are already fully aggregated and remain as such for the rest of the simulation.

The results for the all-atom simulations shown in Fig 10 highlight that aggregation does indeed happen in a short time, as at around 300 ns one unique aggregate can be observed for both NNAT isoforms, and it is maintained for the duration of the simulations.

From the same starting coordinates, two systems were generated: an all-atom system and a coarse-grained one using the SIRAH tool and force field. This choice was made to compare the results obtained by different methods, which could further demonstrate the potential of such techniques to observe protein aggregation.

Notably, in the coarse-grained simulations, even after 4 μs the copies are not fully aggregated, as only smaller aggregates are formed (S9 Fig). This could be due to the approximation introduced by the coarse-graining process, or by the higher friction coefficient used for these simulations. This process could be further studied using additional simulations with different coefficients and different NNAT concentrations, however the all-atom simulations appear to confirm a high aggregation potential for both isoforms, supporting the already available experimental evidence.[3]

## Conclusions

In this study, we aimed to elucidate the structural and functional characteristics of two NNAT isoforms and their interaction with the SERCA2b Ca^2+^ pump. The RosettaFold-generated models, which closely resembled the experimentally solved regulin structures, like PLB and sarcolipin, were used as starting point for MD simulations for refinement. The analysis of the simulation showcased stable conformations, after an initial structural rearrangement, and confirmed a predominant α-helical secondary structure.

To investigate the interaction between NNAT and SERCA2b, we carried out protein∷protein docking using the transmembrane helix of NNAT, as well as *ab initio* AI-based complex generation. The results identified a NNAT binding site within the groove between helices M2, M6, and M9 of SERCA2b, a well-described region known for PLB binding. The consensus reached by several tools supports the hypothesis that the two studied NNAT isoforms can interact with SERCA2b in a manner similar to regulins, potentially modulating the pump activity by altering its conformational dynamics.

Further MD simulations of the NNAT-SERCA2b complexes revealed that both NNAT isoforms influence water flux through the pump, akin to the inhibitory effect observed with PLB. The α isoform showed a slightly greater impact on the passage of water molecules across the membrane, while the β isoform had a greater effect on the formation and length of water wires inside of the pump. Additionally, the formation of a salt bridge between Glu28 of NNAT α and a key residue in SERCA2b suggests specific molecular interactions that could underlie the regulatory effect. The analysis of the stability of the complexes after the phosphorylation of a serine present in both isoforms also highlighted a further similarity with PLB, as this post-translational modification appeared to destabilize the complexes, regulating the inhibitory function of the proteins.

A study of the aggregation potential of both NNAT isoforms using all-atoms MD simulations revealed a notably fast aggregation process. A similar trend can be observed using coarse grained simulations, although at a much slower rate. These results support experimental evidence linking NNAT aggregates to degenerative diseases such as Lafora disease and suggest that coarse-grained simulations might become a viable method to assess protein aggregation for systems that would otherwise be too big for all-atom simulations.

In conclusion, our comprehensive computational findings point out that NNAT isoforms modulate SERCA2b activity via structural interactions similar to those of PLB, suggesting that neuronatin could be considered a “regulin-like” protein. This study widens our understanding of the functional role of NNAT in Ca^2+^ homeostasis and provides foundations for future experimental investigations to explore these regulatory mechanisms and design small molecules affecting NNAT activity.

## Supporting information

S1 File Supporting information file contains: protein::protein docking poses, secondary structure prediction for the two NNAT isoforms, PAE maps, plDDT and Ramachandran plot of NNAT models, alignment of human and rabbit SERCA sequences, pictures of SERCA2b::NNAT complexes, RosettaMPDock results, MD simulation analyses of NNAT isoforms in solution and aggregation propensity in coarse-grained simulations. (DOCX)
