# Supplementary material for: Identification of neuronatin as a SERCA2b regulin-like protein and assessment of its aggregation propensity via coarse grained simulations
Source: PLoS One. 2026 Apr 9;21(4):e0346335. doi: 10.1371/journal.pone.0346335 (PMC13065046; doi:10.1371/journal.pone.0346335)
Supplement: S1 File — (DOCX) [file pone.0346335.s001.docx]

**Identification of neuronatin as a SERCA2b regulin-like protein and assessment of its aggregation propensity via coarse grained simulations**

**Neuronatin: a new regulin-like protein**

Omar Ben Mariem^1*^, Lara Coppi^1*^, Emma De Fabiani^1^, Ivano Eberini^1§†^, Maurizio Crestani^1§^

^1^ Dipartimento di Scienze Farmacologiche e Biomolecolari "Rodolfo Paoletti", Università degli Studi di Milano, Via Giuseppe Balzaretti 9, 20133 Milano, Italy.

^*^ These authors equally contributed to this work

^§^ These authors equally contributed to this work

^†^ Corresponding author: ivano.eberini@unimi.it

**Supplementary tables**

| **Pose number** | **PIPER cluster size** |
| --- | --- |
| 1 | 256 |
| 2 | 222 |
| 3 | 141 |
| 4 | 134 |
| 5 | 97 |
| 6 | 86 |
| 7 | 27 |
| 8 | 18 |
| 9 | 12 |
| 10 | 6 |

S1 Table. PIPER protein∷protein docking poses sorted by cluster size.

**Supplementary figures**

**
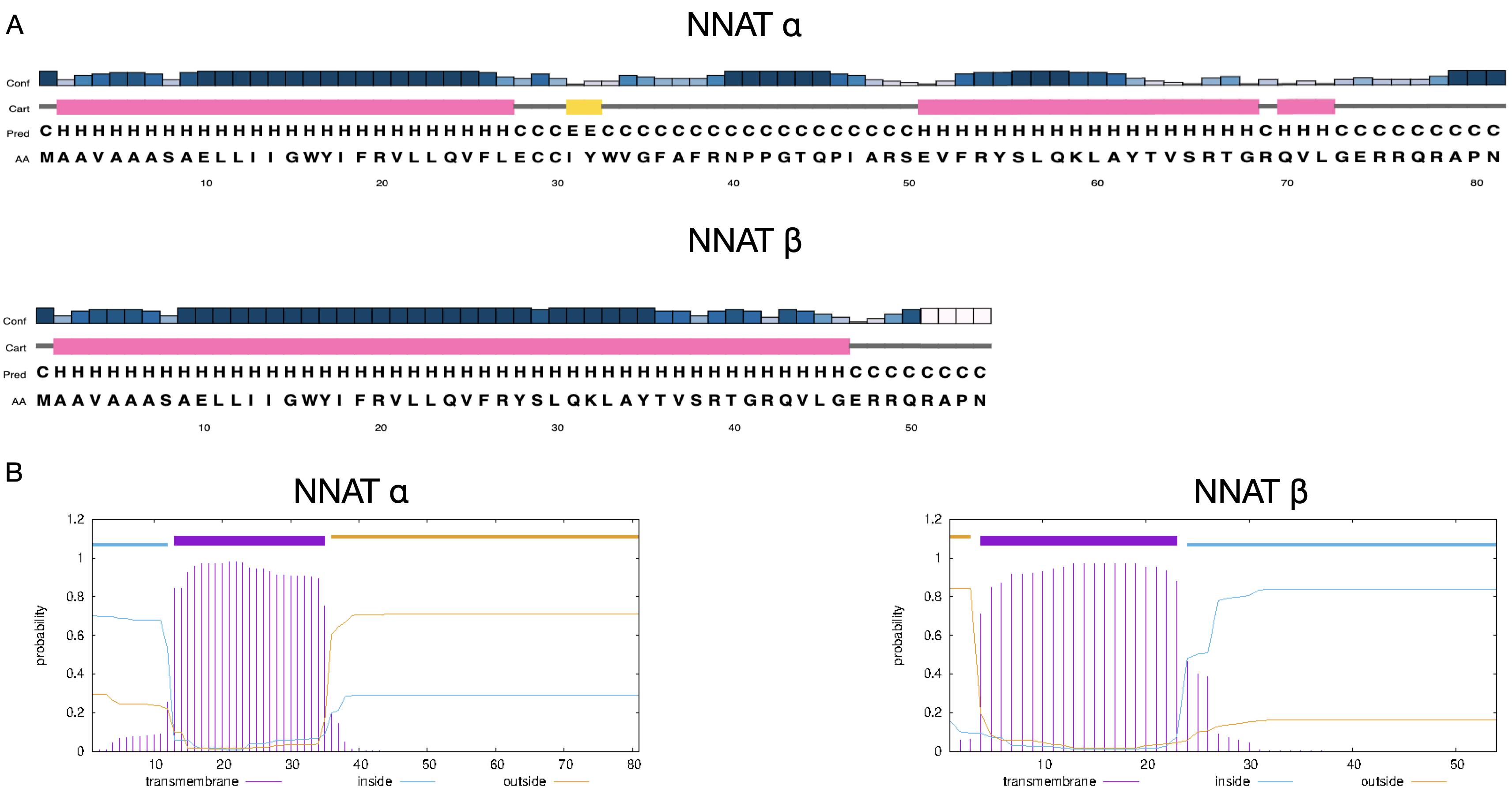
**

S1 Fig. Secondary structure prediction by PsiPred (A) and transmembrane helix prediction by TMHMM (B) for the two NNAT isoforms. All predictions, with small differences, predict the presence of a transmembrane loop at the N-terminal. In the longer α isoform, the two helices are separated by a long unfolded loop, while in the β isoform, no such loop is predicted.


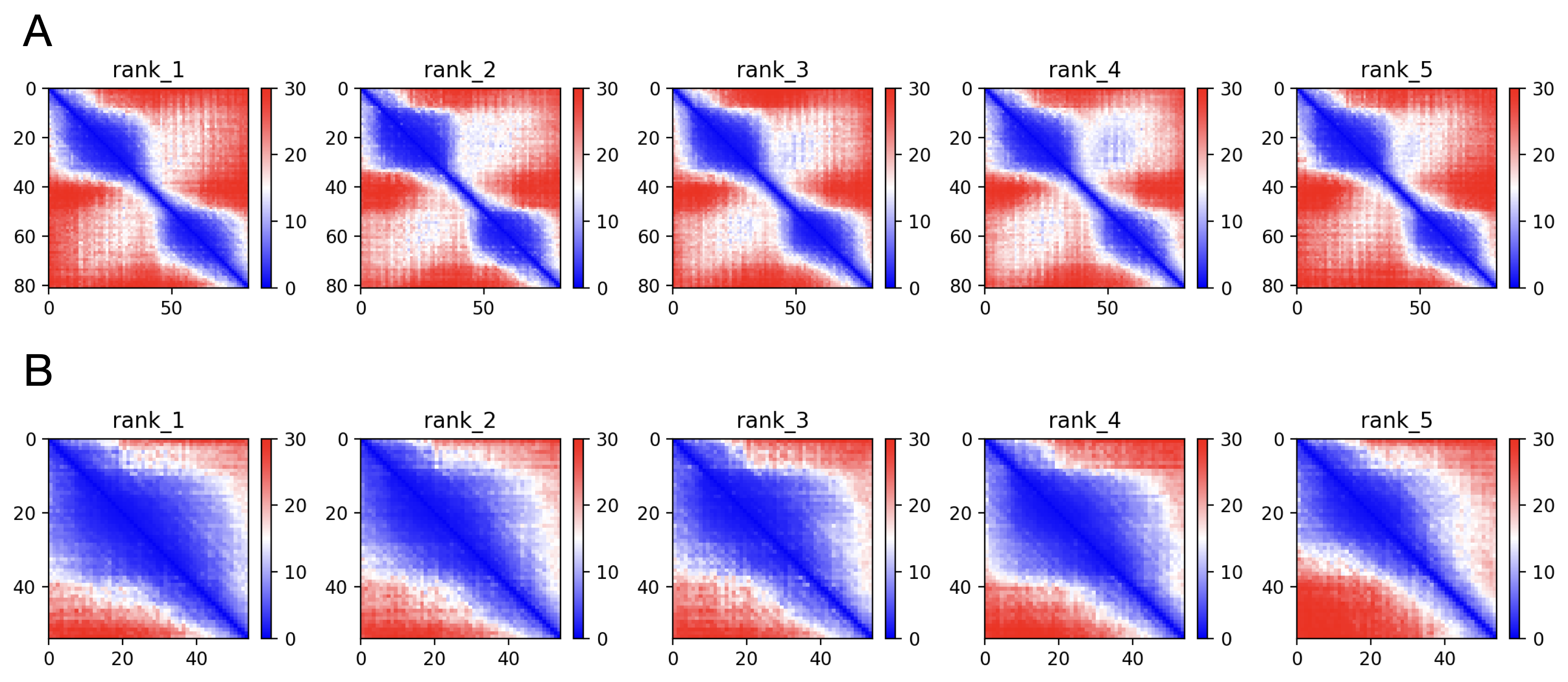


S2 Fig. PAE maps of the Alphafold model of (A) αNNAT and (B) βNNAT


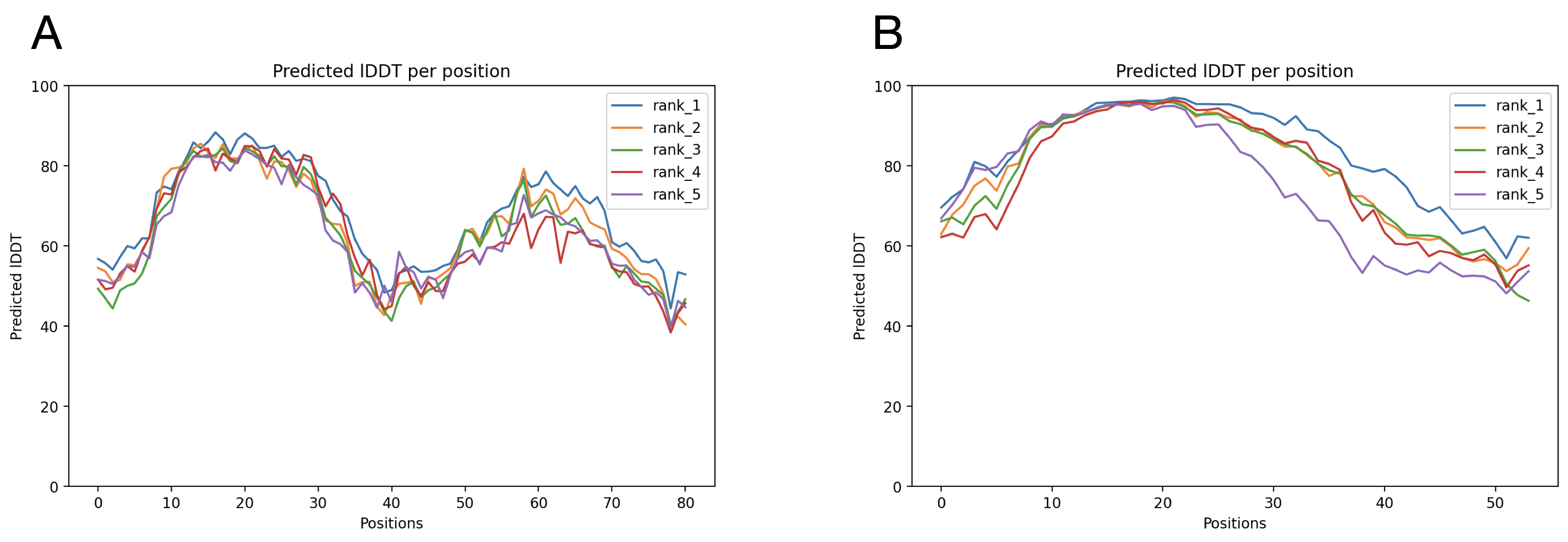


S3 Fig. plDDT graphs of the Alphafold model of (A) αNNAT and (B) βNNAT


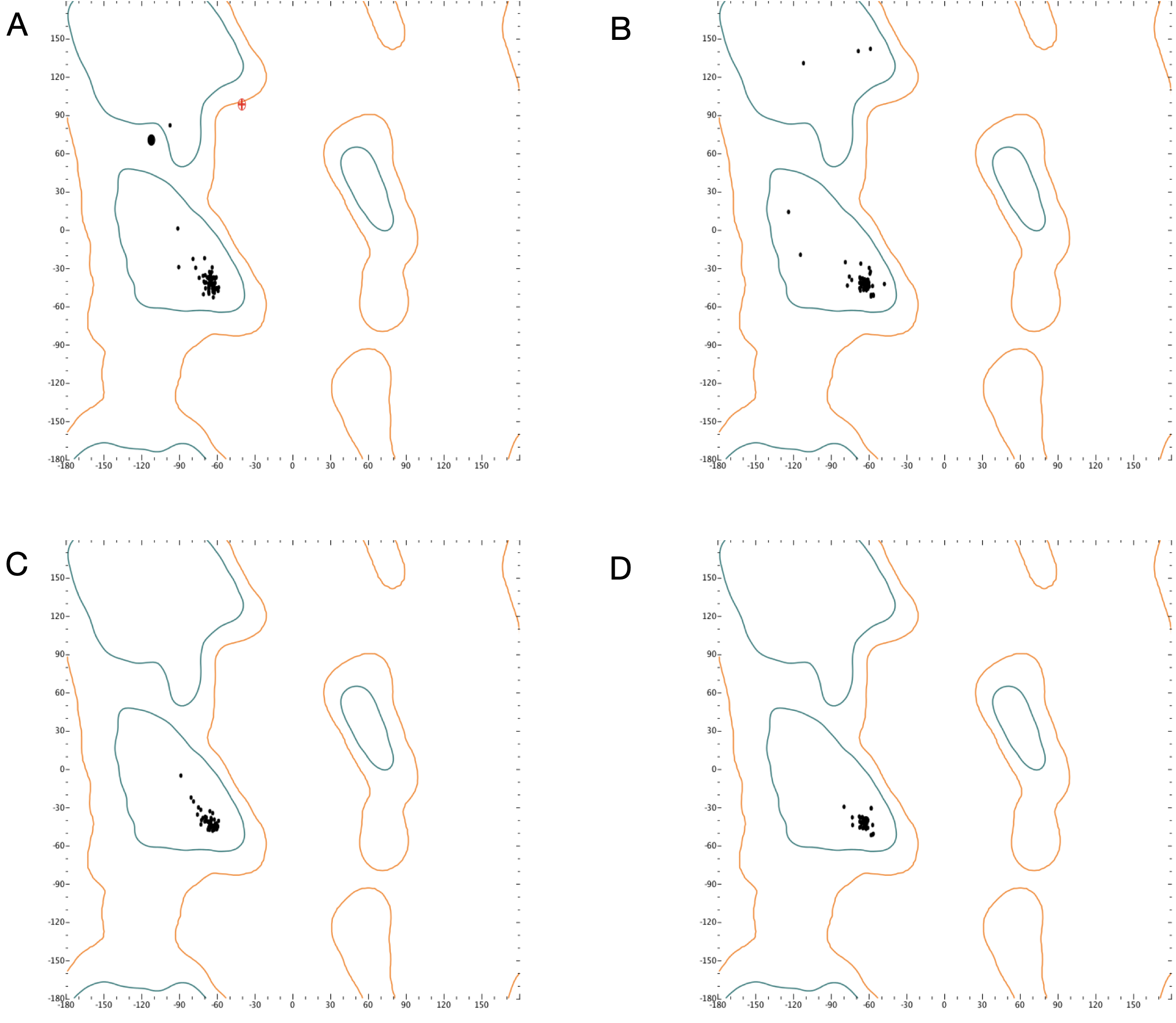


S4 Fig. Ramachandran plot for the AI-generated models. A) NNAT α generated by AlphaFold. One outlier (Arg39) can be observed. B) NNAT α generated by RoseTTAFold. C) NNAT β generated by AlphaFold. D) NNAT β generated by RoseTTAFold

**
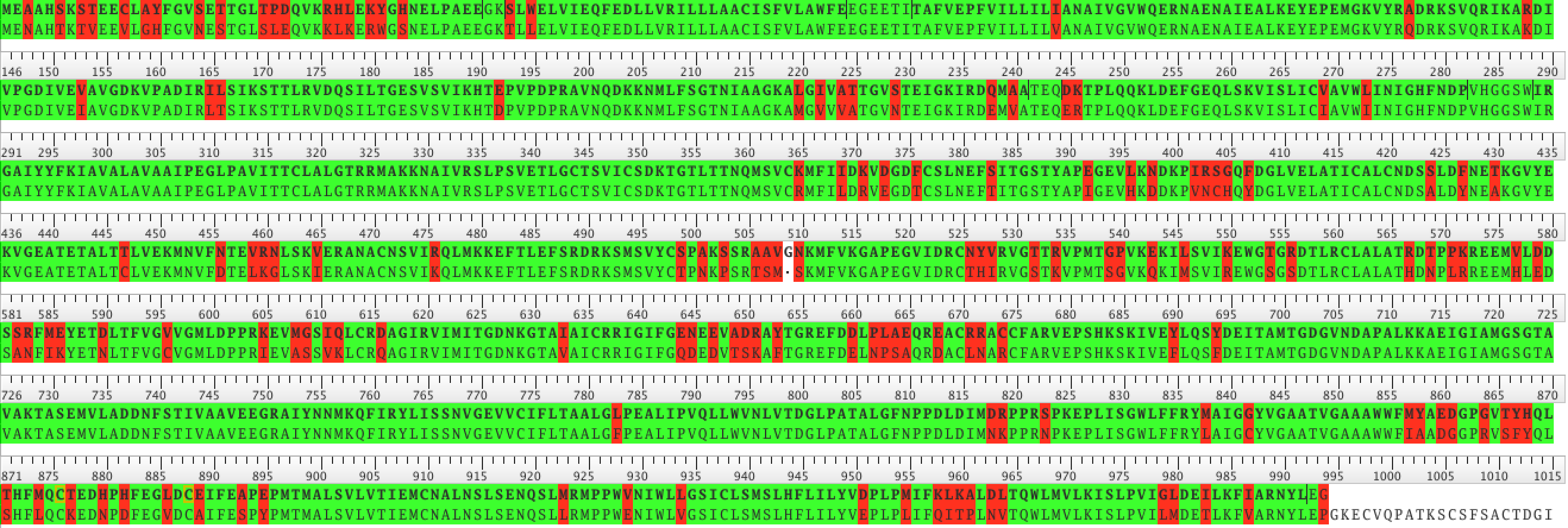
**

S5 Fig. Sequence alignment between the sequence of *O. cuniculus* SERCA1a as present in the crystal structure with PDB code 4KYT and human SERCA2b. The identical residues are colored in green, while the non-identical in red. Even if not identical, most residues are highly conserved (e.g., I→L, D→E, …). ID%=80.2%, similarity % = 89.2%.


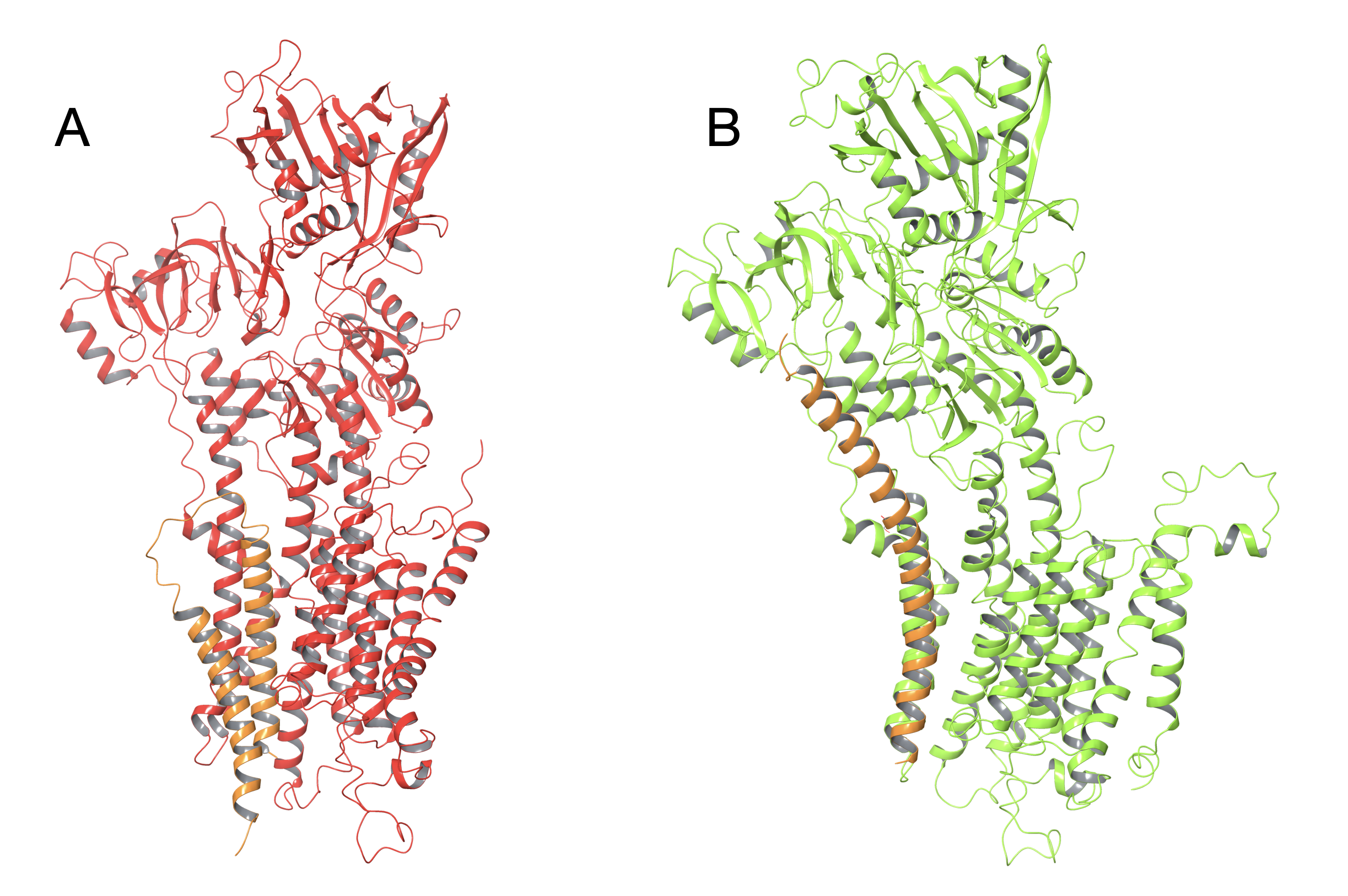


S6 Fig. SERCA2b::NNAT complexes obtained with Alphafold Multimer (A) SERCA2b::αNNAT (B) SERCA2b:: βNNAT.


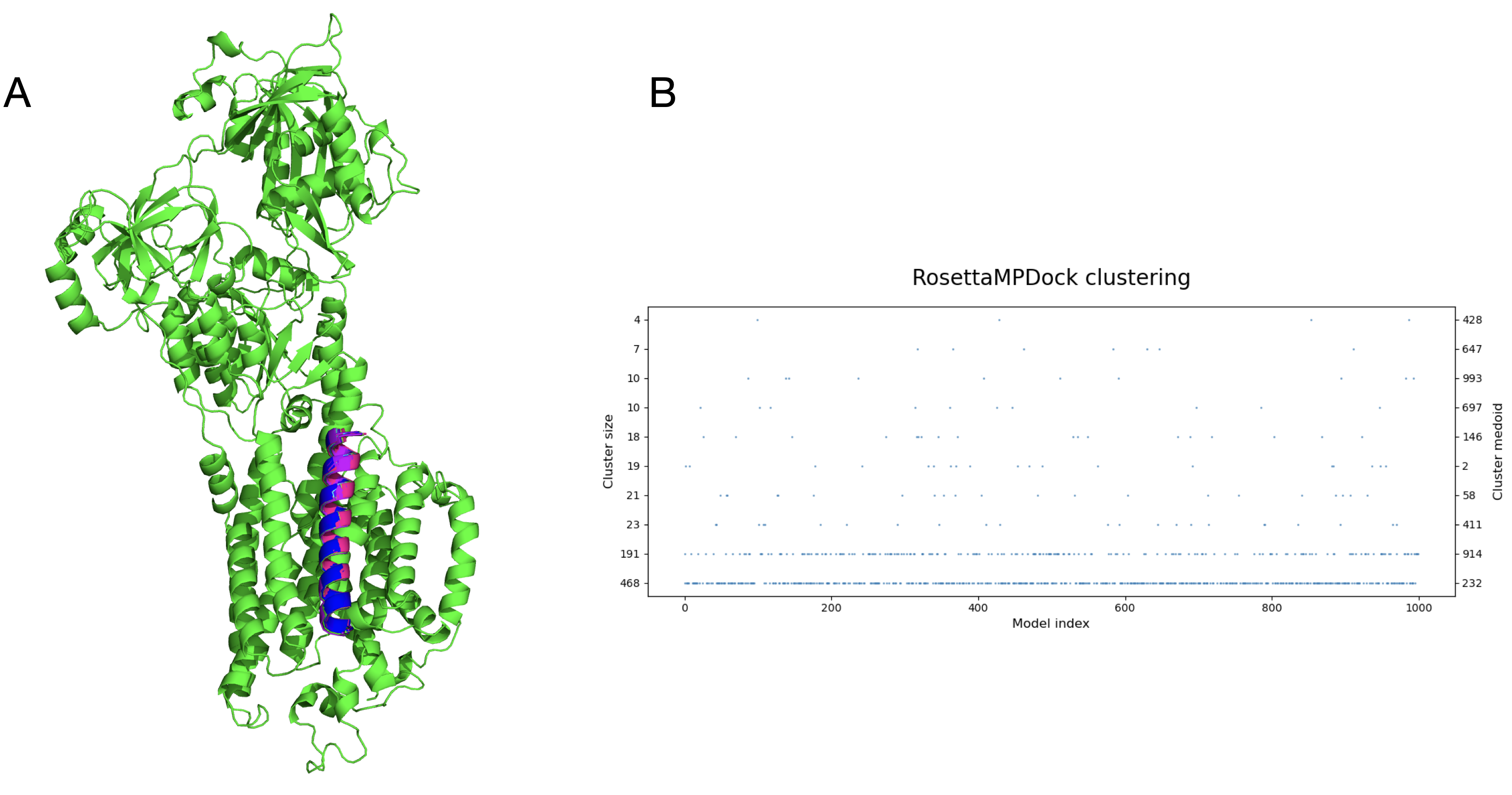


S7 Fig. Results of the docking procedure of the NNAT transmembrane helix on SERCA2b executed with RosettaMPDock (A) 50 best poses, which can be found in the same SERCA2b groove (B) clustering of the 1000 generated poses.


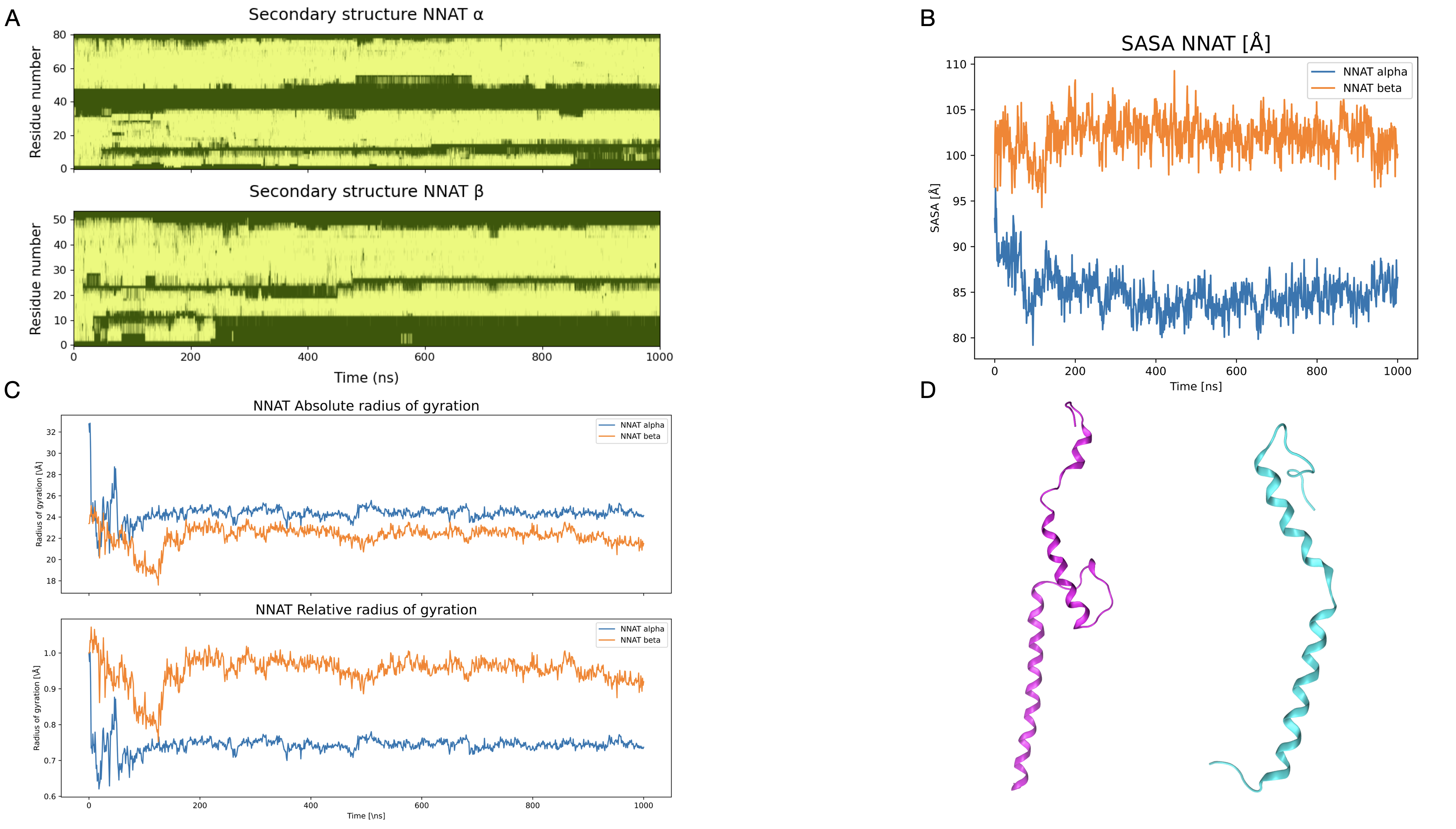


S8 Fig. Analysis of the MD simulations of the two NNAT isoforms in solution. A) Both isoforms tend to lose some secondary structure features, especially at the N-terminal, which is hydrophobic and should be transmembrane. B) SASA of the two isoforms. C) Radius of gyration of the two isoforms, both in absolute and relative values (compared to the first frame). The decrease in radius of gyration is evident for both simulations, but especially for the α isoform, due to its higher degrees of freedom. D) Final structure of the two isoforms (purple: α, cyan: β). For both structures, the N-terminal is at the bottom of the figure.


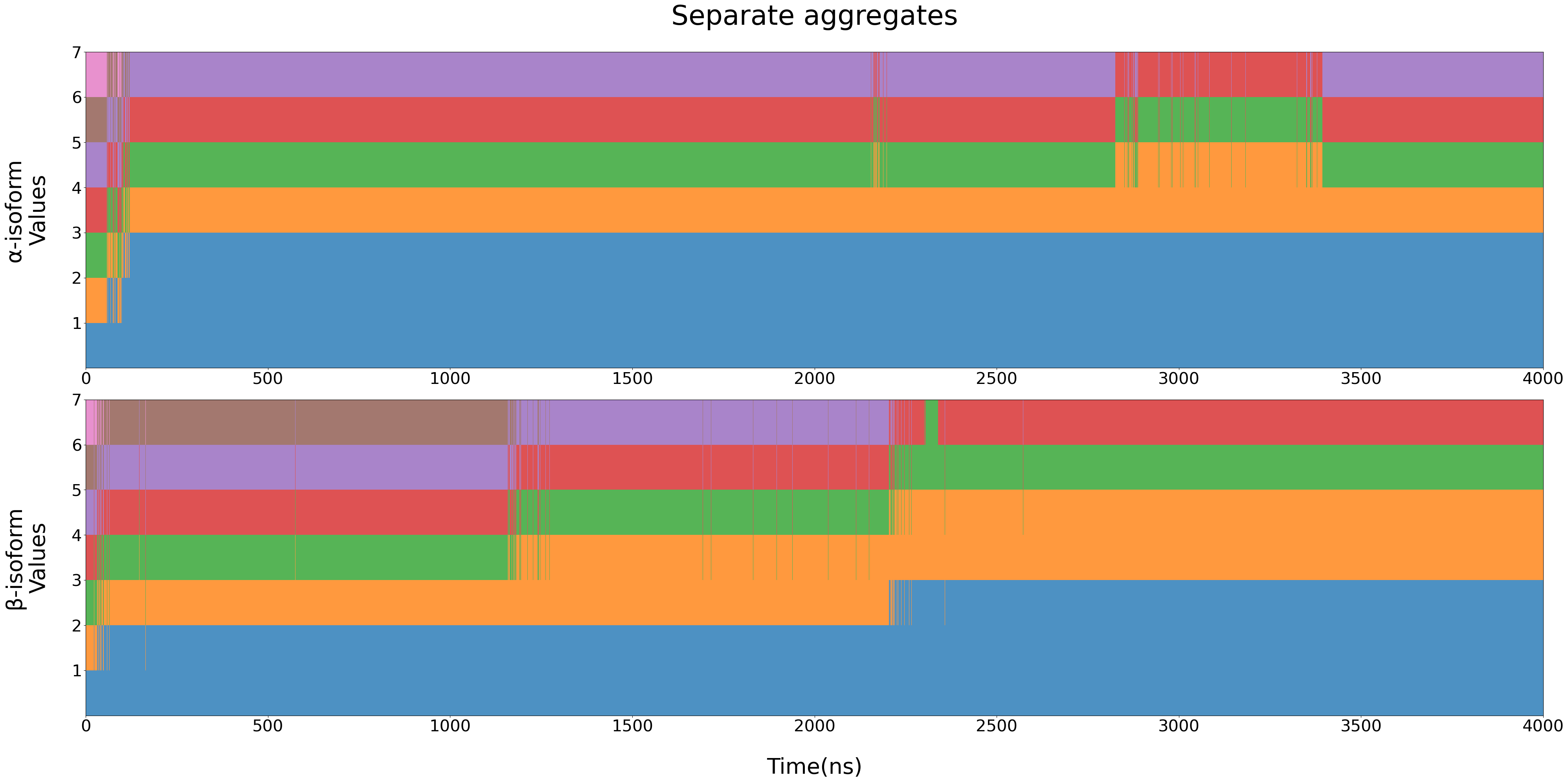


S9 Fig. Separate entities observed for the coarse-grained simulations. Each colour represents a unique “entity”. Differently from the all-atom simulations, it is not possible to observe complete aggregation, even after 4 μs.
